# Modern approaches to address the concept of territorial division in Islamic jurisprudence

**DOI:** 10.12688/f1000research.122335.1

**Published:** 2022-07-18

**Authors:** Hajed A. Alotaibi

**Affiliations:** 1 Department of Islamic Contemporary Studies, College of Science and Humanities at Hotat Sudair, Majmaah University, Riyadh, 11952, Saudi Arabia

**Keywords:** Modern Approaches, Territorial Division, Islamic jurisprudence

## Abstract

**Background**: This article examines how current jurists can deal with a juristic division of the world into abode of “Islam” and “infidelity”. The success with which jurists re-evaluate this dichotomy will have major impacts on how an Islamic state interacts with non-Muslim governments on the one hand, and Muslims residing temporarily or permanently in a non-Muslim territory on the other.

**Methods**: Here, an attempt made to review the concepts of Dar Al-Islam and Dar Al-Harb from two perspectives: that of the dialectical limits and liberalization, as discussed in secondary material such as books, research articles, and reports.

**Results:** this paper has explored and discussed the criteria for identifying Dar Al-Islam and Dar Al-Harb in Islamic jurisprudence.

**Conclusions**: This article analyzes the various arguments put forward to establish new ways of peaceful coexisting and maintaining healthy international connections.

## Introduction

The terms “Dar Al-Islam” (land of Islam or peace) and “Dar Al-Harb” (land of war or blasphemy) have become vital to modern-day Islamic juristic discourse and some Muslim scholars, especially those of the Hanafi schools such as Alkasani (2010) and Alsarkhasi (2001), who divide the entire world into Dar Al-Islam and Dar Al-Harb. In addition, others (e.g. Alzuhaili, 1981) have added a third term, “Dar Al-Ahd” (house of truce or treaty), to the existing Islamic division of the world between Dar Al-Islam and Dar Al-Harb. Arguably, the introduction and use of this third term is a response to the many circumstances and events that have emerged which challenge the old Islamic binary discourse. For example, there have been political developments in matters of state sovereignty in the public arenas of international law, social change, and geographical movements and affiliations. Ongoing immigration has caused a crucial reconsideration of the Dar Al-Islam/Dar Al-Harb divide because, for example, Muslim immigrants have been required to adapt themselves to new and different realities (i.e. new countries, new peoples, multiple religions, etc.) in order to apply for citizenship or for a passport from that country and so on. There has been considerable focus on immigrants’ children and the extent to which they adapt themselves to such challenges or conserve their original culture, religion, and languages within their new communities.

The issue of immigration has led to some other crucial questions arising that have required a further critical review of the old juristic division between Dar Al-Islam and Dar Al-Harb. For example, can the concept of Dar Al-Islam still exist while the state of Islam is divided into multiple countries (i.e. it is not a single state/empire as in the past)? Can the non-Muslim world be described as a place of war, while Muslims find more protection and security in the West than their Islamic countries? Is it possible to invest some of the juristic opinions into developing a broader concept of homeland, thereby facilitating the idea of coexistence, and creating an understanding between those who differ in religion and residency?

Alshirazi (2018) claims that the relationships that once existed and prevailed between countries, regarding the concepts of sovereignty, the minority, and the majority, are no longer the same in this era in which treaties and agreements have a prominent impact on the international reshaping of the United Nations. Additionally, advanced and advancing information technology have impacted on the reshaping of international relations, with technology also having a strong impact on the spread of information, and transmitting ideas and beliefs to people around the globe regardless of objections, barriers, or issues pertaining to rights/permission.

In contemporary times, interactions between the countries of the world are largely based on mutual commercial benefits, political and military collaborations, as well as mutually agreed uses of social spaces, all of which are enacted independently of belief and ideology. Given these contemporary international transformations and formations, it is arguably not tenable for contemporary jurists to posit a juristic division of the world based solely on the dichotomy of “the house of Islam” and “the house of infidelity”. It follows, given this, that there is a question as to whether they will adhere to this division or respond to the changes of their era. The response has serious ramifications for the nature of Islamic countries’ relationships with non-Muslim governments on the one hand, and Muslims living temporarily or permanently in non-Muslim territories on the other.

## Methods

In order to assess the data suitability, the author applied certain criteria. For instance, I searched using the keywords mentioned above, to find out in Google Scholar and in Saudi Digital Library SDL, which has access via our institutions to major research databases. Also, access to more than 169 Databases including ProQuest, Emerald, Web of Science, Scopus, Taylor & Francis and many others. Additionally, SDL has access to more than 50,000 journals around the globe and more than 5,000,000 postgraduates’ theses and dissertations. Therefore, after specifying those secondary research data, the author has prepared the required discussion using qualitative approach, dividing them into sections based on thematic titles as shown throughout the paper. For example, breaking the discussion into its appropriate places of the paper, trying to look at both sides of an argument so as to overcome the alleged bias, if any, in the paper. Moreover, based on secondary data such as books, research articles, and reports, this paper reviews the concepts of Dar Al-Islam and Dar Al-Harb from two perspectives: those of dialectical constraints, and liberalization. It additionally examines the Islamic division of the world through the old Islamic juristic discourse by examining the criteria for defining Dar Al-Islam and Dar Al-Harb in Islamic jurisprudence. The material from this preliminary discussion is then used to suggest new ways of peaceful coexistence as well as the development of good international relationships.

## Results

### Land of Islam and land of war in old Islamic juristic discourse

Sergeevich (2020) traces the evolution of the concepts of Dar Al-Islam/Dar Al-Harb in Arabic dictionaries as well as Islamic jurisprudence in order to examine their development. He concludes that the concept of a world divided into two geo-religious territories emerged in the early centuries of Islam, that the terms represent the distinction between the Muslim and non-Muslim worlds, and that they are the standard used to decide who belongs to both of those realms. It is also the case, however, that the terms also possess resonances of cultural symbols and values. Huwaidi (1990) observes that some Muslim jurists, in the past and present, have divided the world into two major parts. Others such as Alzuhaili (1981) have added two additional divisions, “Dar Al-Ahd” (land of the covenant or treaty) and “Dar Al-Hyad” (land of neutrality). As Huwaidi (1990) points out, dividing the world into two or three named sectors is not just the invention of Muslims, even if Muslim jurists adopted and developed it. The Romans, for example, divided people into Patriots, Latins, and Foreigners, with “foreigners” originally being called “enemies”, “others” or “barbarians”. Huwaidi argues that the Romans came to adopt this latter way of thinking due to the rise of Islam at that time. Indeed, the Romans began to use the parallel religious concepts of “Christian homes” and “homes of disbelief”.

In Islamic juristic discourse, however, more complex criteria are applied to those lands. Indeed, there is no single basis that can be considered to define the lands of Islam, of war, or of covenant, even though a search will reveal various definitions of those terms. Alzuhaili (1981) argues that there is no such thing as a “Dar Al-Hyad”(land of neutrality) in Islam since it is included in “Dar Al-Ahd” (the land of the covenant). This is because the concept of the land of the covenant has been expanded to cover all non-Muslim countries in the world today, as long as infallibility remains and fighting does not exist. Peace is the foundation of Islamic international relations. Ahmad (2008) additionally cites Alzuhaili’s argument that the legal repercussions of an act done in such a state (i.e. Dar Al-Hyad or land of neutrality) are rarely discussed in Islamic jurisprudence. This may be because such a state did not exist in the immediate vicinity of the jurists in question or, more crucially, because the inhabitants of such a state did not have the legal protection of the Islamic state due to the absence of a treaty. For a variety of reasons, the old Islamic juristic texts have defined the lands of Islam, Harb, and Ahd in various ways. However, from the following definitions, it is evident that Muslim jurists differ in their definitions of the lands due to their differences of opinion about the standards and controls set out for them. Definitions are largely based on the following criteria:

1) The criterion of worship (the ability to demonstrate the two testimonies – that Allah is the one God and that Mohammed is his prophet – and the Pillars of Islam, such as prayer, zakat, and so on).
2) The criterion of authority and governance (the rule of Islamic law or lack thereof).
3) The criterion of military power (i.e. whether the military power supports a benign Islamic government or is at the disposal of other militant groups).
4) The criterion of the size of the population, whether large or small.

As a result, Muslim jurists have given “Dar Al-Islam” several definitions, and these include that of Alsarkhasi (2001, p1253) who notes that Dar Al-Islam is a name for the place that is in the hands of Muslims, a sign of which is that Muslims feel secure in it. Meanwhile Al-Kasani (2010, p131), of the Hanafi school, has written that: “What is meant by adding the houses of Islam and disbelief is not Islam or disbelief in themselves. Rather, what these terms describe are security and fear, respectively. Consequently, it means that if there is safety for Muslims at all times, then it is the abode of Islam. Otherwise, it is the abode of unbelief”.

Abu Mansur Al-Baghdadi of the Shafie school (1980, p270) argues that: “Dar Al-Islam is every house (territory) in which the call of Islam has arisen from among its people without a guard, judge or tribute”. He further observes that, “Muslim law covers the people of the dhimma (non-Muslims with protected status) if the people of the heresy did not fight against the people of Sunnah, for it is the abode of Islam”. He concludes, “If the matter is against what we have mentioned, then it is the abode of unbelief”. Meanwhile, Ibn Muflih of the Hanbali School (2003, p190) defines the differences between Dar Al-Islam and Al-Harb as follows: “Every land that has been conquered by Muslim armies and that has a Muslim government is the land of Islam, but if the rule of the infidels prevails, according to Islamic teachings, then the land is the land of Al-Harb. There are no other additional houses”.

According to the Zaydi sect of Shia Muslims, “Dar Al-Islam is where the two testimonies and prayer appear, and where there is, additionally, no blasphemy, except in the eyes of Muslims” ( Alshawkani, 2004, p57). He argues that when Jews and Christians practice their religion in Muslim countries this is not deliberately anti-Islamic. It is simply that such religious practices offend Muslims because they deviate from Islam.

The definitions of contemporaries who cling to the old juristic division of the lands do not deviate from the terminology of the ancients. For instance, such scholars see that Dar Al-Islam is every country in which Islamic law prevails in legislation and implementation, and that power and pride are with Muslims, whether the majority of the population are Muslim or not. As for the house of war, this encompasses all lands in which there is no sovereignty or protected status for Muslims, where Islamic judgments in law are not applied, and where Muslims are not able to live under an actively Islamic government ( Abu Zahra, 1995, p277; Afifi, 1986, p128; Alzuhaili, 1981, p175).

From the discussion above, it becomes clear that Muslim jurists differ in their definitions of Dar Al-Islam, Dar Al-Harb and other terms used to describe the relationship between Muslim and non-Muslim lands, territories, and governments. However, Ahmad (2008) argues that the central distinction between Al-Islam and Al-Harb has been widely misinterpreted in recent years. It is commonly conceived that the partition of the universe into two domains is inextricably linked to the belief that the usual relationship between Dar Al-Islam and Dar Al-Harb is uniformly one of enmity. In light of such comments, it is frequently asserted that one must either embrace or reject the following two assertions:

• That the globe can be divided into a “Land of Islam” and a “Land of War” and
• That the relationship between Muslim and non-Muslim countries is necessarily one of hostility.

Those scholars who see the two assertions as inextricably linked argue that the divide envisioned by Muslim jurists is permanent and unchangeable. This may, in turn, lead to violent thinking. However, according to Bonzatto and Ortunes (2015), fundamentalism is a recent but not always violent movement. Commenting further, it should be noted that Bonzatto and Ortunes (2015) examine the concept of a house of peace and a house of war, and note various interpretations. Fundamentalism, according to Bonzatto and Ortunes, “presents the threads coming from a period of conflict and reform in Islam.” As a result, each fundamentalist group has its own interpretation of the Qur’an, as well as its own political and religious goals. However, for a better knowledge of the subject, it is necessary to rely on some books by authors who hold opposing viewpoints, such as Bernard Lewis and Edward Said.

Badar and Nagata (2017) look at how the terms Dar Al-Islam and Dar Al-Harb have evolved throughout Islamic history, and note how extremist groups have misinterpreted them, and what their current legal standing is. Badar and Nagata concluded that modern extremist groups have resurrected the use of some older Islamic doctrines and intentionally misconstrued them to achieve their goals. They opine that the most prominent example of such a group is Daesh (the Islamic State of Iraq and Syria). Daesh has issued takfir (excommunication) pronouncements against Muslim governments, and claimed that only Daesh-controlled territory is Dar Al-Islam, while all other Muslim countries are Dar Al-kufr (having a Muslim population but not governed by Islam). Meanwhile, to explore why that Daesh is the only Dar Al-Islam, scholars such as Bernard Lewis (2004, p73) wrote that the universality of the Muslim revelation is the foundation of the requirement for jihad. It is the obligation of those who have accepted God’s word and message to struggle unceasingly to convert or, at the very least, subjugate those who have not. This responsibility has no time or space constraints. It must continue until the entire globe has either joined Islam or succumbed to the Islamic state’s sovereignty. Until then, the world is divided into two houses: the house of Islam (Dar Al-Islam), where Muslims govern and Islamic law reigns supreme, and the house of war (Dar Al-Harb), where the law of war – or the potential for Muslim conquest – reigns supreme.

Those who do not agree that the typical international relationship between Muslim and non-Muslim political entities is one of conflict claim that Muslim jurists drew this distinction based on the conditions of their time, and that it is unsustainable in present times. As contemporary thinker Tariq Ramadan (1999) argues, Dar Al-Islam and Dar Al-Harb are notions that are not found in either the Qur’an or the Sunnah, whose ideas are global and eternal, and transcend all geographical boundaries. In contrast, during the first three centuries of Islam, Islamic scholars evaluated and classified the many places in and around them at that time. For example, they looked at the geographical divisions, the powers in place through religious affiliation and influence, and the ever-changing nature of alliances.

The practice of dividing the world into two domains (Islam and Harb) has been retained primarily by the Hanafi School; a majority of Muslim schools such as the Maliki, Shafie, and Hanbali have rejected this division. Therefore, the question arises as to what motivated Hanafi scholars to establish such a division and why most other Muslim scholars have rejected it. According to the Hanafi School, Muslims who live in Dar Al-Islam are protected (ma’soum) by Islamic law. For Muslims living outside of Dar Al-Islam, protection merely means that those who abuse their rights will be held accountable in the Next World, i.e. in the hereafter, by God’s court. This is known as “Ismah bi’l Islam,” or protection based on one’s association with Islam ( Alsarkhasi, 2001).

According to Hanafi jurists, this means that the rights of Muslims living outside Dar Al-Islam’s territorial bounds will not be enforced by the Islamic state’s courts, as these courts can only exercise jurisdiction over the area under the effective control of Dar Al-Islam’s Imam (i.e. the ruler). In other words, Islamic courts are unable to take recognition of a judicial action that occurs outside of their jurisdiction. The Islamic state’s courts do not have jurisdiction over wrongs committed outside the borders of the state unless it has a contract or treaty with the body that has suzerainty over that territory. In contrast, the Islamic state is obliged to preserve the rights of all those who dwell permanently, or even temporarily, inside its borders, whether Muslim or non-Muslim. All people living within an Islamic state’s geographical boundaries are assured of legal protection, whether Muslim or non-Muslim. This is known as the “protection of one’s rights as a result of residing in an Islamic country” ( Alsarkhasi, 2001).

The bottom line of the Hanafi School’s position is that “Ismah” (used here according to its core meaning of “protection”) guaranteed solely to those who live inside the geographic bounds of the Islamic state, regardless of whether they are Muslims or non-Muslims, according to what we may call, the municipal law of Islam. Furthermore, Islamic law differentiates between various non-Muslim political bodies based on their attitude toward Islam and Muslims. If a non-Muslim country is actively at war with Dar Al-Islam, it is referred to as Dar Al-Harb whilst if it signs a peace treaty with Dar Al-Islam, it is known as Dar Al-Ahd. However, people living in Dar Al-Harb do not enjoy ‘Ismah’ (legal protection) from the standpoint of the Islamic state’s territorial authority and its courts ( Alkasani, 2010). In instance of Dar Al-Ahd, however, and because the Islamic state has signed a treaty with that entity, certain jurisdiction may be ceded to the Islamic state. If this is the case, some sort of “Ismah” may be established for individuals who live there. The provisions of the treaty that has been signed will determine the status of the previously hostile country and its inhabitants.

In contrast to the Hanafi School, the majority of Shafie, Maliki, and Hanbali Muslims believe that Islamic law has no territorial boundaries. As a result, if a Muslim disobeys a Shariah norm, he will be punished not only in the Hereafter but also in this life by the Islamic state’s courts. Similarly, if a non-Muslim resident of a non-Muslim state abuses the rights of any Muslim, the wrongdoer will be punished by the Islamic state’s courts if he is captured by Muslims or enters the Islamic state. It follows, that “Ismah,” or legal protection, is conferred because of a person’s religious affiliation (Islam) rather than his or her domicility in a politically determined land (Dar). Concerning non-Muslim citizens of an Islamic State (Ahl Al-Dhimmah), it should be noted that it is believed that they are protected as long as Muslims have signed a Dhimmah treaty with them. A majority of Shafie, Maliki, and Hanbali Muslims thus compare non-Muslims to alien non-Muslims (musta’minn) in Dar Al-Islam; protected by Muslims under an Aman (reciprocal safety) contract ( Ibn Qudamah, 1999; Ibn Alqayim, 2014).

Tariq Ramadan’s (1999) assertion that the division of the world into Dar Al-Islam and Dar Al-Harb does not exist in the Quran or Sunnah is open to challenge, especially about Sunnah literature. His un-nuanced assertion may have led to confusion about this matter. The next section of this paper discusses some Islamic juristic rulings including some from the basic sources of Islamic law, on the Islam/Harb distinction, from a territorial (as opposed to purely religious) perspective.

### Discussion of territorial division in Islamic jurisprudence

Ahmad (2008) argues that though we cannot always deduce directly the teachings of the Islamic texts (i.e. the Quran and the Sunnah), certain things may be inferred. Thus, instead of interpreting the texts literally, Hanafi scholars such as Alkasani and Alsarkhasi strive to extract general principles from these texts and then apply them to the entire field of law. If a ruling emerges from one of the most authoritative texts that is different from the literal interpretation given in a text of lesser authority, they interpret the secondary text in light of the general principle. This may result in them treating the rule mentioned in the secondary text as an exception to the general principle if compatibility between the two is impossible. Through this approach, the Hanafi preserve uniformity in the judicial system.

Passages and traditions, ranging from inferred principles from the Quran to explicit directives in both the Quran and other kinds of Islamic literature, establish different laws for Muslims who live in and beyond Dar Al-Islam. For example, the Qur’an classifies some Muslims who immigrated from Makkah (at that time Dar Al-Harb) to Medinah (at that time Dar Al-Islam) as “fuqara” (poor; empty-handed) even though they owned property in Makkah (Qur’an 59: 8). According to Hanafi scholars, the reason for this interpretation was that they had lost the titles to their property in their native land because of their move to Dar Al-Islam. Another proof of their loss of ownership was the fact that the Prophet Muhammad did not return these assets to them after the conquest of Makkah ( Hamidullah, 1987; Alsarkhasi, 2001).

When the Muslims in Makkah were persecuted and forced to flee to the Islamic state of Medinah, those who did not migrate were denied the Islamic state’s protection (Quran 16: 106–110 and 4: 97–99). The Quran thus asserts that the Islamic state (Dar Al-Islam) has no legal responsibility to protect the rights of such people. However, if requests for assistance are received regarding “matters of faith,” the Islamic state is obliged to assist those people, even militarily, if necessary (Quran 8:72; Quran 4: 75–76). It is also the case that the Islamic state is compelled to act according to the conditions of any treaty they sign. Further comment on the territorial nature of the Islam/Harb divide is found in the case of Abu Busair and his associates. These people were not subject to the Islamic state’s control since they were Muslims who had immigrated to places other than Medinah. However, the Prophet did not feel legally obliged to hand them over to the non-Muslim rulers of Makka either. It is worth noting that, as a non-Hanafi scholar, Ibn Alqayim (2014), a well-known Hanbali jurist, sees grounds for a territorial jurisdiction in this case. He claims that by not putting a halt to these people’s activities, the Prophet did not break any treaty provisions because they were “out of his control”. In short, they were not under his jurisdiction.

Additional evidence of the Islam/Harb divide as a territorial matter includes the idea, at least according to Hanafi jurists, that non-Muslims can acquire ownership of property they capture from Muslims if they carry it to Dar Al-Harb. Several arguments have been advanced in support of this rule. It is important to note, however, that taking possession of property by force does not provide a justifiable reason for its ownership within Dar Al-Islam. According to the Hanafi school of thought, this is because the basis for “Ismah” (legal protection) is Hirz (safe custody), which is based on Dar (a land’s jurisdiction) rather than Din (religious belonging).

However, what of the territorial Islam/Harb divide and monetary exchanges? According to Abu Hanifah and Muhammad Ibn Al-Hassan Al-Shaibani, if a Muslim exchanges one Dirham for two Dirhams in Dar Al-Harb, the transaction is valid, because the Muslim exchange partner took the property with his or her consent. The matter of a Muslim’s property in Dar Al-Harb is unlike that of the property of the musta’minn (Non-Muslims with a protection contract) in a territory of Islam. Musta’minn property becomes legally protected under the contract of Aman (reciprocal safety) ( Alsarkhasi, 2001 p.104)

Moreover, if a Muslim soldier commits adultery or fornication (Zina) in Dar Al-Harb, he will not receive the appropriate punishment because the cause of action occurred beyond the Islamic state’s jurisdiction. However, if he commits such an act within the Muslim army’s camp, additional punishment can be imposed. The same is true when it comes to imposing the Qisas (death) penalty ( Alsarkhasi, 2001 p. 84, 115, 116)

Correspondingly, the courts of Dar Al-Islam are unable to resolve any musta’min issues (issues concerning non-Muslim temporary residents of the Dar Al-Islam) that emerged while they were still in Dar Al-Harb. The musta’min do not become residents of Dar Al-Islam just by having a contract of Amn. However, they also need to be in Dar Al-Islam territory at the time that the issue arises if legal redress is required.

Muslims should still respect a person’s rights if he comes to Dar Al-Islam from Dar Al-Muwda‘ah (a territory of mutual peace) without a new Amn contract. Similarly, if he enters Dar Al-Harb and Muslims control the land thereafter, Muslims must maintain his rights even there, “his status is similar to that of a dhimmi who joins a Dar Al-Harb that is later controlled by Muslims.” ( Alsarkhasi, 2001 p.98). The above examples are from Hanafi scholars’ points of view.

In contrast, other Muslim scholars, including the Maliki, Shafie, and Hanbali schools are of the view that Islamic law is applicable everywhere, especially, to Muslims as well as to those who are in contract with Dar Al-Islam, though this depends on the contents of their contracts. They argue that the legal protection “Ismah” of the faithful Muslim relates to a Dar Al-Islam based on the universal religion and not merely one of physical territory. According to this perspective – and with reference to the example previously given - those Muslim immigrants who lost the titles of their property in their native land as a result of their move to Dar Al-Islam, were entitled to have their property returned once Makkah was conquered by Muslims, as happened later on in history. Those Muslims who did not migrate from Dar Al-Harb were entitled to the Islamic state’s protection ( Ibn Qudamah, 1999; Ibn Alqayim, 2014).

Moreover, if the “location” of Dar Al-Islam and Islamic law can be anywhere where there are Muslims, non-Muslims cannot acquire the ownership of property that they may capture from Muslims if they carry it to Dar Al-Harb. What is more, if a Muslim exchanges one dirham for two dirhams in Dar Al-Harb, the transaction will be void because Muslims are bound by Islamic law everywhere. It is also the case that if a Muslim soldier commits adultery or fornication (i.e. Zina) in Dar Al-Harb, he must submit to the appropriate punishment. Similarly, the courts of Dar Al-Islam can oversee and resolve any musta’min issues that emerged whilst the plaintiffs were still in Dar Al-Harb but only once the issue has been raised in Dar Al-Islam. It remains the case, however, that the situation regarding Abu Busair and his associates is not applicable to the concept of Dar Al-Islam as a religious concept rather than one of geographical place. The Prophet Muhammad’s treaty with the Makkans included a provision requiring him to return anyone who migrated to Medinah, where he then lived. Abu Busair and his associates immigrated to a different place from Medinah (in other words, they moved outside the geographical bounds of the treaty). Thus, the Prophet did not break any treaty provisions Ahmad (2008).

The discussion above makes clear that both major views emanating from the Islamic schools of jurisprudence are inaccurate. This is because the division of the world into two parts, whether framed in religious or territorial terms, is juristic; hence, it lacks both legal and Sharia reasoning, although the division itself is arguably based on the divine texts of the Quran and Sunnah. Yet this division is purely derived from received wisdom rather that constant rational argument. Eqigg (2017) claims that continuing to adopt the old juristic view of the concepts of “house of Islam” and “house of war” can lead to unfortunate results. For instance, in the mentality of terrorist movements, it could provide justification for aggressive acts directed against non-Muslims in their lands, whether as combatants or civilians. In addition, a too simplistic division of the world into Islam and Harb may cause extremists to harm Muslims residing in non-Muslim countries. Furthermore, by advocating the juristic dictionary definitions of homelands, the jurists may encourage some Muslims in the West to abrogate the money and properties of non-Muslims by enabling them to argue that it is permissible to deceive the private and public facilities of some countries on the pretext that since their inhabitants live in a “house of war/unbelief”, the property is free for Muslims. In doing so, they make two grave errors:

First, they are flouting the legal and Sharia rulings that urge the fulfillment of pledges and the keeping of trusts and charters - even where these have been entered into with people who profess a different religion. The second is that they substantiate the negative image of Muslims that may already exist in non-Muslim countries.

### Land of Islam and land of war in contemporary Islamic juristic discourse

Contemporary Muslim jurists such as Aljudai (2007), Alhaj (2006) and Alfaitori (2006) approach the concept of an Islam/Harb divide from several angles in order to analyze and evaluate this heritage according to what is consistent with the Qur’anic vision of civilized living. In addition, Bork (2017) discusses modern views of the territorial division between the Lands of Islam and War in ancient Islamic legal theory, and in so doing focuses on views that challenge obsolete constructs of the world and advocates appropriate substitutes. He does this by utilizing the perspectives of two modern Muslim thinkers: Tariq Ramadan and Wahbah Al-Zuhail who have both attempted to establish new models which consider present realities, as well as offering a vision of Muslim-Non-Muslim cooperation based on Islamic law. The perspective of many contemporary jurists can be summarized as follows.

First, they emphasize that the issue is generally perceived through the lens of custom and usage rather than according to binding legislation. Moreover, based on the criteria referred to above, contemporary juristic writings unanimously state that the division of the world into “Dar Al-Islam”, “Dar Al-Harb” and “Dar Al-Ahd” is the work of the jurists in the second century of the Prophet Mohammed. Alhaj (2006) observes that such a division arose from, “the necessities and conditions imposed by the special societal circumstances at that time”. This juristic development of the original sources is not based on significant legal evidence but rather, on, well-intended interpretation. It should not, therefore, be taken as a binding religion, but rather as a guide. Moreover, Ian argues that Dar Al-Harb, in particular, could have been “civilized” in the same way that the Byzantine Empire had been, but that it had not bowed to God’s will as defined by Islamic theology and jurisprudence. It is also worth noting that this terminology is fluid and has evolved over time, with varying degrees of consensus among scholars and rulers about what it actually indicated in terms of geographical region.

Some contemporary researchers such as Alali (2006) have attempted to establish this juristic division by citing a group of hadith texts in which the terms “Dar Al-Islam” or “Dar Al-Hijrah” are mentioned, and their opposites, “house of polytheism” or “house of infidelity”. They argue that such texts confirm that this division was common in the words of the Companions. However, whilst these references can possibly be interpreted as describing a divided world, they are not definitive. Meanwhile, the context in which the terms “Dar Al-Hijrah”, “house of the faithful” and “house of immigrants” mentioned are in the context of a doctrinal and faith dimension, which has nothing to do with the political significance that later jurists added to it. Whatever the motives and purposes that led those jurists, either from Hanafi or from other Islamic schools, to make this division; they biased their interpretation of it more towards faith and belief than towards the politics of a geographically conceived Islamic nation ( Alhaj, 2006). Furthermore, their interpretation of the concept does not describe either an original or a permanent basis for the geography of the land of the Islamic caliphate, which represents a starting point for all perceptions about the nature of internal and external relations, particularly, for the Muslim community. Secondly, contemporary jurists such as Al-Qaradawi (2010) and Topolyak (1997) have employed a new concept of “safety” to emphasize that the “house of Islam” at present is no longer confined to the historical boundaries of the old “house of Islam”. Rather, they suggest, it has expanded greatly to include every spot in which there is a Muslim who acknowledges to his Lord the two testimonies and establishes his laws in himself according to his ease and ability. Badr (1982) claimed that historical Islam’s international connections have gone through three periods of varying duration: the ages of expansion, interaction, and coexistence. Any attempt to define Islamic international relations regulation must keep this historical context in mind.

Suleiman Muhammed Topolyak, a modern Islamic researcher, records in his discussion of the ruling on the political asylum of a Muslim in a Non-Muslim country that the concept of ‘abode of infidelity’ has changed today from what it meant in the past. For instance, we can see that a foreigner in a non-Muslim country enjoys legal and judicial protection, which are the same as a citizen, regardless of a religion. Yet, in many cases, these immigrant Muslims are allowed to practice their religious rituals, establish mosques and Islamic centers without objection ( Topolyak, 1997). In the same way, Al-Qaradawi (2010) states that a Muslim may reside in any country in which the environment of religious, political, and civil freedom prevails, even if it is a secular country. Especially, if immigration or residence in those countries is for legitimate purposes such as seeking sustenance, knowledge or asking for security.

By referring to the old juristic tradition, we find such words among Alshafie (2001), where he says: “the Messenger of God said: the imposition of emigration can only be on those who afford it, but those who have been tempted in their religion in a country where they embraced Islam. Because the Messenger of God authorized people in Makkah to stay there in Makkah after their conversion to Islam such as Al-Abbas bin Abdul-Muttalib because they did not fear sedition". Ibn Taymiyyah (2005) believes that the criterion of either residing in the abode of infidelity or emigration is based on the extent to which a Muslim can obey God in that abode or not. Therefore, there is no doubt that this can only be achieved with the presence of security and safety. If a Muslim find this in a place where he lives, then there is no need to emigrate. This is the all-encompassing principle".

To explain more, the command to emigrate in Islamic law is related to the existence of safety elements in religion, self, and accommodation. However, with the conquest of Makkah, the necessity to emigrate was no longer necessary as it was a base of Islam and, additionally, safety exists there. Therefore, emigration is to a place that achieves for a Muslim the purpose of emigration, which is to enable him to establish his religion and protect himself from harm. This indicates that staying among non-Muslims is not self-blameworthy. Rather, it depends upon the extent of what is achieved from it in the interests of the Muslim. When contextualizing some Hadiths in this regard, confirming the legitimacy of the residency of a Muslim in other than the abode of Islam, including the survival of a group of companions who immigrated to Abyssinia and did not join the Messenger of God in the house of Islam, i.e. Medinah, during the seventh year of the Hijra. The Prophet did not blame them for immigrating to the abode of Islam nor did he issue anything that offended their stay among non-Muslims in Abyssinia ( Eqigg, 2017). It was previously stated in the early juristic definitions of Dar Al-Islam that the Hanafis make a criterion of distinguishing Dar Al-Islam from the other houses by “achieving security and safety for Muslims”. This safety can be achieved according to Zaydi jurists by the ability of just showing the two Shahada and some rituals of Islam ( Alshawkani, 2004, p575; Alsarkhasi, 2001).

Third, the specific political and social data of the world has changed. The juristic classification of homelands, as previously mentioned, is a discretionary classification dictated by the conditions of the Islamic nation and the nature of international relations existent at the given time. However, the situation has changed, and the existence of recognized international treaties, the criminalization of wars that have not arisen from a response to aggression and resistance to occupation, and the emergence of states of citizenship, as well as the rights of religions, races, and colors necessitate that that the whole world has become a space for tolerance and peaceful coexistence between and among all human beings. Henkel (2004) conducted an empirical study in Germany and concluded that many religious Muslims have recently undergone a major shift to a more “inclusive” position. To understand this transition, Henkel studied the transnational experience of Turkish Muslims in Germany, especially the experience of the “second generation” of Turkish immigrants, as well as the recent development of Turkey itself, where Islam has accelerated its integration into modern society. As Henkel notes, for many religious Muslims of the traditional Turkish Islamic tradition, a free society has become a social background conducive to the practice of Islam.

Commenting upon associated themes, it can be seen that some of the analysis of the Orientalist Bernard Lewis is based on the classic Islamic comparison between Dar Al-Islam and Dar Al-harb. In his reading, he shows the inherent hostility of Islam to non-Muslims. However, the conflict is only one aspect of the complex relationship that exists between Islam and “Western” societies. For most Muslims, Dar Al-Islam and Dar Al-Harb are no longer relevant categories that define their social relationships with non-Muslims. However, the conceptual opposition of Dar Al-Islam and Dar Al-Harb points to a question that remains important to religious Muslims and that has been answered in different ways at different times: in what kind of society can one live a Muslim’s life? According to Arangul (2017), millions of Muslims presently live under non-Muslim governance. This is a level of magnitude unmatched in the past. Muslims were unwilling to legitimize non-Muslim supremacy for a variety of reasons during periods when they had free and/or prosperous countries. However, when historical reality begins to turn against them politically and economically, immigrant Muslims have grown to believe that this is feasible if they follow certain Islamic teachings.

Moreover, the “house of Islam”, whose standards were defined by old jurists, no longer exists except in historical and juristic works. The abode of Islam became distributed among many nations of more than fifty countries, and its names became linked to races and nationalities as well as, sometimes, the names of families and royals (i.e. not in the name of the religion itself, which was the basis for belongings). In addition, whereas Muslim people of the old abode of Islam did not leave unless they were merchants, involved in Jihad or as tourists or visitors to other places for limited periods, today, they are in many parts of the world in which Muslims permanently reside, either through long-standing affiliations to those regions or by action.

Given these new determinants, Mawlawi (1990) believes that the criteria that the jurists specified in the past to distinguish the house of Islam from the house of war are no longer valid for defining an accurate description of this house in this era. Accordingly, he poses the questions: What is the standard to the Sultan of Islam, what are the implementations of its provisions, and what is needed to establish its rituals? Is it the establishment of Islam completely? This means that most Muslim countries are no longer a “house of Islam” today ( Haikal, 1996). Is it sufficient to apply the provisions of Islamic personal status to the exclusion of all other laws? This means that we exclude the “house of Islam” of ancient Islamic countries like Turkey and Tunisia. Is it sufficient for Muslims to freely perform the rituals of Islam such as prayer, fasting, Hajj and zakat to be considered a “house of Islam” based on the legacy of the past? There are Muslims and they practice their rituals more freely than in some Muslim countries Mawlawi (1990). As for the “house of war”, it is no longer clearly defined, given that Muslims are now linked with most of the countries of the world that are under the banner of the United Nations with treaties, charters, and alliances that cannot be revoked as long as everyone abides by their requirements per what is customary in “international legitimacy” Mawlawi (1990).

In addition to this, the “house of war” - according to Howeidi (1990) is no longer necessarily located in the square of others. Rather, most wars in this time take place in the homes of Muslims; a scenario that did not occur in the time of the ancient jurists, as they only envisioned war between the Muslims as a whole, and the non-Muslims as a whole. Then “our jurists who used the term Dar Al-Harb did not live the terrestrial unity that we live in today, but they lived in a world of separate islands which do not coexist, hence, it was the jurisprudence of war.

Ahmad (2008) wrote that the Quran and Sunnah texts do not lend themselves to such an extension of the abode of Islam and war because, like all past Messengers of God, the Prophet’s wars included an element of Divine punishment for his foes, and he had, therefore, conclusively demonstrated the reality of his mission beyond all doubt, at least as far as his immediate addressees in seventh-century Arabia were concerned. Other than the Prophet’s closest addressees, non-Muslims cannot face any worldly punishment for their rejection of Islam. This is because it would violate the Quranic prohibition on compulsion in questions of religion (Qur’an 2: 256). This part of the Prophet’s mission was also acknowledged by the fuqaha, who used it as the foundation for various regulations. In any case, Jihad does not advocate for the coercive conversion of non-Muslims to Islam. Rather, Muslims must oppose those who seek to disrupt the Divine plan by harassing those who seek to force them to accept or reject a certain faith.

Therefore, what we need today is the “jurisprudence of peaceful coexistence”; a reality that is different in quantity and quality. Mawlawi (1990) also claimed that the West is no longer a house of battle, but rather a house of call because the premise on earth is that it is for the call, based on the Almighty’s statement that “We did not send you but a mercy for the worlds”. Moreover, jurisprudence councils in Europe, America, and India have unanimously agreed that the land of Muslim minorities is a place of covenant and call, not a place of conflict ( Al-Awa, 2006). It is critical, therefore, to be cautious when words from old Islamic international law such as “Dar Al-Islam,” “house of infidelity,” “house of war,” and “house of covenant,” to the realities of today’s world and individual countries as a consequence of the changed nature of international relationships, their complexities, and the nature of overlapping attributes of the same.

## Conclusion

“Dar Al-Islam” and “Dar Al-Harb,” have become dangerous phrases in Islamic jurisprudence. However, several circumstances have emerged that put into doubt Dar Al-Islam and Dar Al-Harb in classic Islamic discourse. State sovereignty, immigration, geographical mobility, and citizenship are all effective examples of such ongoing issues. As a result, Muslim jurists have been compelled to adjust to a new and different reality (new countries, people, religion, language, and so on). As a result, new approaches to the old Islamic jurisprudence’s concept of geographical partition have, and are, being developed. It follows that some legal opinions could be invested to expand the concept of homeland, promote peaceful coexistence, and resolve the issue of religious and residency differences. Issues pertaining to how an Islamic state interacts with non-Muslim governments on the one hand, and how Muslims reside temporarily or permanently in non-Muslim territory, have been influenced by this debate. The concept of Dar Al-Islam and Dar Al-Harb, as well as the criteria for identifying Dar Al-Islam and Dar Al-Harb in Islamic jurisprudence have been examined in this work and new path for the furtherance of peaceful coexistence and healthy international relationships identified.

## Data availability

No data are associated with this article.
