# Supplementary material for: Modern approaches to address the concept of territorial division in Islamic jurisprudence
Source: F1000Res. 2023 Aug 10;11:794. Originally published 2022 Jul 18. [Version 3] doi: 10.12688/f1000research.122335.3 (PMC10636343; doi:10.12688/f1000research.122335.3)
Supplement: Peer review history

# Reviewer report: Reviewer response for version 3

**DOI:** 10.5256/f1000research.154204.r195436

**Published:** 2023-11-09

**Authors:** Che Zarrina Sa’ari

**Affiliations:** 1 Academy of Islamic Studies, University of Malaya, Kuala Lumpur, Malaysia

The article "Modern Approaches to Address the Concept of Territorial Division in Islamic Jurisprudence" delves into a critical and timely exploration of the traditional Islamic divisions of the world into "Dar Al-Islam" and "Dar Al-Harb" and the contemporary challenges posed by factors such as immigration, state sovereignty, and technological advancements.  The article presents a clear distinction between "Dar al-Islam" and "Dar al-Harb," emphasizing that they are concepts related to the regulation of laws and safety rather than actual physical locations. This distinction is well explained and contributes to a better understanding of the terms.

 The historical context provided about the emergence of these concepts during the early Abbasid dynasty, in response to conflicts between Muslim and non-Muslim nations, is informative. It offers valuable insight into the motivations behind the categorization of states into "Dar al-Islam" and "Dar al-Harb." The article highlights how some contemporary scholars advocate for more inclusive and cooperative approaches that transcend the rigid boundaries of Dar Al-Islam and Dar Al-Harb. It discusses the role of safety, security, and freedom in determining whether a place can be considered a "land of Islam" and the idea that Muslims can reside in non-Muslim countries under specific circumstances, such as seeking sustenance, knowledge, or security.

 Moreover, the article recognizes that the traditional division of Dar Al-Islam and Dar Al-Harb has become less relevant in the current geopolitical landscape due to changes in international relations, the prevalence of treaties and alliances, and the existence of Muslim minorities in non-Muslim-majority countries.

 The article suggests that instead of relying on the traditional terminology of Dar Al-Islam and Dar Al-Harb, contemporary scholars and Muslims need to adopt a "jurisprudence of peaceful coexistence" that acknowledges the complexity of today's world and individual countries. It emphasizes that the concepts of the "land of peace" and the "land of war" are not explicitly based on the Quran and Sunnah and were introduced later in Islamic history to address specific historical and political contexts.

 In conclusion, the article provides a comprehensive review of how contemporary Muslim scholars are reevaluating and challenging the classical division of the world into Dar Al-Islam and Dar Al-Harb. It reflects the ongoing debate within Islamic jurisprudence about how these concepts should be understood and applied in today's globalized world.

 However, it's essential to note that this review is **largely descriptive and expository. **It provides an overview of various scholars' perspectives on this topic **but does not engage deeply with critical analysis or provide an overarching argument**.

 Thus, several aspects require further consideration and refinement:

 The article could benefit from improved organization and clarity. The introduction introduces key terms and concepts, but the transition between sections could be smoother. **A clearer delineation between the introduction, methodology, and the main content of the study is essential**.

 Methodology: The article mentions using certain criteria to assess data suitability but lacks explicit details about the research methodology, **especially on data analysis.** A **more in-depth explanation of the research methods of data analysis **would enhance the scholarly rigor of the study.

 Depth of Analysis:

 While the article touches upon several intriguing topics, it could benefit from **a deeper analysis of specific case studies, historical examples, or recent events that illustrate **the challenges and changes in the conceptualization of Dar Al-Islam and Dar Al-Harb.

 The article refers to various scholars such as Abou El Fadl and their perspectives on the terminology. However, it would be academically beneficial to **provide specific citations or references to these scholars' works for readers to explore further**.

 Overall, the article provides a well-structured and informative analysis of the concepts of "Dar al-Islam" and "Dar al-Harb" in Islamic jurisprudence, their historical context, and their relevance in the modern world. To enhance its academic rigor, the article should **consider providing more specific citations and references to support the claims made, ensuring that readers have access to the scholarly sources that underpin the arguments presented**.

 Original Contribution: The article claims to present highly advanced ideas, yet it is essential to clarify how these ideas are unique and distinct from existing literature. **Highlighting the novelty of the research findings and their contribution to the field is crucial for academic relevance**.

Is the work clearly and accurately presented and does it cite the current literature?

Partly

If applicable, is the statistical analysis and its interpretation appropriate?

Not applicable

Are all the source data underlying the results available to ensure full reproducibility?

Yes

Is the study design appropriate and is the work technically sound?

Yes

Are the conclusions drawn adequately supported by the results?

Partly

Are sufficient details of methods and analysis provided to allow replication by others?

Partly

Reviewer Expertise:

Islamic thought

I confirm that I have read this submission and believe that I have an appropriate level of expertise to confirm that it is of an acceptable scientific standard.

# Reviewer report: Reviewer response for version 3

**DOI:** 10.5256/f1000research.154204.r205643

**Published:** 2023-09-13

**Authors:** Ramadhita Ramadhita

**Affiliations:** 1 Universitas Islam Negeri Maulana Malik Ibrahim Malang, Malang, East Java, Indonesia

This article has explained the views of Islamic jurists on the concept of territorial division and peace building in the world. This article has referred to the latest studies. This article deserves to be indexed.

Is the work clearly and accurately presented and does it cite the current literature?

Yes

If applicable, is the statistical analysis and its interpretation appropriate?

Partly

Are all the source data underlying the results available to ensure full reproducibility?

Yes

Is the study design appropriate and is the work technically sound?

Yes

Are the conclusions drawn adequately supported by the results?

Partly

Are sufficient details of methods and analysis provided to allow replication by others?

Partly

Reviewer Expertise:

Islamic law; family law

I confirm that I have read this submission and believe that I have an appropriate level of expertise to confirm that it is of an acceptable scientific standard.

# Reviewer report: Reviewer response for version 2

**DOI:** 10.5256/f1000research.140000.r177261

**Published:** 2023-07-18

**Authors:** Ramadhita Ramadhita

**Affiliations:** 1 Universitas Islam Negeri Maulana Malik Ibrahim Malang, Malang, East Java, Indonesia

I think this paper is excellent and is an important addition to the literature. The author should add an explanation that this research is a quantitative research with a conceptual approach. The data obtained is based on secondary data and classical to modern Islamic law books. The data obtained is compared and contextualized with the current conditions.

 Introduction:

- The author needs to convey the urgency of the research, there is not much literature cited in this introduction. The author has not conveyed the argumentation and novelty aspects of this research - the social unrest and the evidence.

Methods:

- Did the researcher use the systematic literature review method in this study? If yes, then SLR has a special method related to the relevance of the article taken. If using SLR then the title should also be adjusted. If not SLR, then you must provide a detailed explanation of what your data sources are, how many were taken, the basis for taking references, how to process, and checks on the adequacy of the data.

Discussion:

- What are the legal implications of the current dichotomy and addition of state models from the perspective of Islamic constitutional law. Which countries have adopted the Hambali school of thought as the basis of their constitutions?

References:

- It's about 70 articles if using SLR.

Is the work clearly and accurately presented and does it cite the current literature?

Yes

If applicable, is the statistical analysis and its interpretation appropriate?

Partly

Are all the source data underlying the results available to ensure full reproducibility?

Yes

Is the study design appropriate and is the work technically sound?

Yes

Are the conclusions drawn adequately supported by the results?

Partly

Are sufficient details of methods and analysis provided to allow replication by others?

Partly

Reviewer Expertise:

Islamic law

I confirm that I have read this submission and believe that I have an appropriate level of expertise to confirm that it is of an acceptable scientific standard, however I have significant reservations, as outlined above.

# Response

**Published:** 2023-07-19

**Authors:** Hajed A. Alotaibi

**Affiliations:** Majmaah University, Saudi Arabia

Thank you for providing me with such constructive advice and insights. I will revise the paper and re-submit it again. However, I just have some responses regarding some points of the review that the fourth reviewer asked me for as follows:

 "The author should add an explanation that this research is a quantitative research with a conceptual approach. The data obtained is based on secondary data and classical to modern Islamic law books. The data obtained is compared and contextualized with the current conditions."

 Actually, the article is not a quantitative research, but rather, it is qualitative research based on secondary data such as books, articles etc. Therefore, no need for a literature review nor SLR because this how it works with qualitative data since there is no statistical data obtained in the paper.

# Reviewer report: Reviewer response for version 2

**DOI:** 10.5256/f1000research.140000.r183641

**Published:** 2023-07-17

**Authors:** Natana Delong-Bas

**Affiliations:** 1 Associate Professor of the Practice, Boston College, Boston, Massachusetts, USA

The article overall is vague in terms of historical timeframes. The legal concepts of Dar al-Islam and Dar al-Harb were introduced at particular moments in time (as noted in a broad reference to the Abbasid Empire), but were subject to significant development in the legal literature over time as empires and nations have encountered different situations. Many of the issues broadly addressed here are covered in detail in Tariq Ramadan's work on Western Muslims and the Future of Islam, particularly relevant classical and contemporary legal approaches. I am concerned to see a work by Robert Spencer in the bibliography given his lack of academic credentials.

 I think the article simply tries to cover too much territory for an article. Some of the descriptions of the scholars under discussion sound like they were taken from a Wikipedia page and do not reflect familiarity with the content, such as Abou El Fadl. It is not clear to me why the author relies only on secondary literature rather than engaging the rich Arabic language Islamic legal material to trace developments on the topics under study. Many of the assertions about the division of the world into 2 spheres were applied during the age of formal Empires. Some would argue that this age has passed and that the transition into an age of nations and nation-states requires an alternative framework, particularly in light of global commercial interconnections which were mentioned at the opening. Given Saudi Arabia's significant legal developments in commercial law, it is surprising not to see any mention of this.

 Essentially, this article presents a review of some literature, but the structure and argumentation are confusing. I'm not sure how Romans qualify as Muslims? I also see no mention of Dar al-Sulh. There are a variety of legal parameters developed over time to permit peaceful relations between different countries.

Is the work clearly and accurately presented and does it cite the current literature?

Partly

If applicable, is the statistical analysis and its interpretation appropriate?

I cannot comment. A qualified statistician is required.

Are all the source data underlying the results available to ensure full reproducibility?

No source data required

Is the study design appropriate and is the work technically sound?

No

Are the conclusions drawn adequately supported by the results?

No

Are sufficient details of methods and analysis provided to allow replication by others?

Partly

Reviewer Expertise:

Islamic law, Islamic studies, Jihad

I confirm that I have read this submission and believe that I have an appropriate level of expertise to state that I do not consider it to be of an acceptable scientific standard, for reasons outlined above.

# Response

**Published:** 2023-07-19

**Authors:** Hajed A. Alotaibi

**Affiliations:** Majmaah University, Saudi Arabia

Thank you for providing me with such constructive advice and insights. I will revise the paper and re-submit it again. However, I just have some responses regarding some points of the review that the third reviewer asked me for as follows:

 I think the reviewer forgets that the author is of Arabic native tongue. Thus, he has consulted various Arabic literature on the matter. Additionally, the article is not a quantitative research, but rather, it is a qualitative research based on secondary data such as books, articles etc. Therefore, no need for a literature review neither SLR because this how it works with qualitative data since there is no statistical data obtained in the paper. 

 Specifically, my research has indicated that some of the current legal-political views or problems are based or depend on the distorted perceptions caused by such terms of Dar Al-Islam and Dar- Al-Harb embedded in almost all the previous conducted researches, as detailed throughout the paper via the literature review. However, in response the comments made by the reviewer, it is useful to summarise and examine some of what the literature review suggested in this regard.

 For instance, Alrousan (2010) conducted a discussion on the "judicial individualization of the punishments" that involved investigating the likely settings for inconsistency in any criminal verdict including various matters such as where the criminal committed the crimes (is it at Islamic or non-Islamic country). The distinctiveness of that work has been long-lasting mainly because it recognizes a number of criteria for assessing punishments in general (not just for Muslims and Non-Muslims, adults or juveniles male or female etc). He concluded that punishment is a necessary evil that must acknowledge an awareness of its negative impacts and also its expected educational role and so reduce the recourse to the punishment. The primary purpose of this is the ability to develop techniques to identify appropriate penalties to re-integrate offenders into society, re-educate them, persuade them to respect societal values and to inform them of their duties. Therefore, it is possible to rely on age, gender, behaviour and the psychological elements of the criminal apart from the place/country where the crime is committed). This knowledge is very different from what the traditional Islamic legal schools (e.g. Hanafi, Hanbali) called for because it is not only about the external circumstances of the crime and the offenders' previous convictions. This was the case for some of the previous Muslim scholars; however, while this argument was revealing, it is not sufficient in itself. This is because, unlike Alrousan, I am not trying to develop a generic profile of trade or criminal verdicts. Rather, my aim is to set out a common profile of the subject including different juristic examples as detailed in the paper. With this purpose in mind, I needed to focus on diversifying the examples presented, analysing and examining them to alert current and upcoming generations of the importance that this topic has in its implications and applications.

# Reviewer report: Reviewer response for version 1

**DOI:** 10.5256/f1000research.134312.r144595

**Published:** 2022-09-06

**Authors:** Che Zarrina Sa’ari

**Affiliations:** 1 Academy of Islamic Studies, University of Malaya, Kuala Lumpur, Malaysia

1. The author should further explain that this juristic division of the world is used/practiced in political matters or dividing borders in the contemporary world and give evidence to support this claim. Specify the states or countries that practice this division especially from Hanafi jurists.
2. If the author cannot prove that this juristic division of the world is used/practiced to show the borders or states, and that it has only been discussed in the jurists' books, what is the importance of this issue being discussed in this article? Please provide convincing arguments.
3. Add more current sources (within 5 to 10 years back) to support your data.
4. The author cited many quotations from many scholars/books that show his weakness in dealing with and analyzing collected data. Thus, he could not show his own understanding of the issues discussed.
5. The terms used are not consistent, please relook to the translation of the term Dar al-Islam, Dar al-Harb and Dar al-Ahd.
6. This article aims to identify and introduce new approaches to expand the concept of homeland, promote peaceful coexistence, and resolve the issue of religious and residency differences as well as develop a new path forward for peaceful coexistence and better International relations. However, the author only highlighted them in the conclusion without identifying, examining, outlining, and discussing specific new paths. 

This article can be said to be written in the form of a review paper and very little highlights its own ideas in building a new idea to face the juristic division of the world.

Is the work clearly and accurately presented and does it cite the current literature?

Partly

If applicable, is the statistical analysis and its interpretation appropriate?

Not applicable

Are all the source data underlying the results available to ensure full reproducibility?

Yes

Is the study design appropriate and is the work technically sound?

Yes

Are the conclusions drawn adequately supported by the results?

Partly

Are sufficient details of methods and analysis provided to allow replication by others?

Partly

Reviewer Expertise:

Islamic thought

I confirm that I have read this submission and believe that I have an appropriate level of expertise to confirm that it is of an acceptable scientific standard, however I have significant reservations, as outlined above.

# Reviewer report: Reviewer response for version 1

**DOI:** 10.5256/f1000research.134312.r144597

**Published:** 2022-08-08

**Authors:** Chris PreJean

**Affiliations:** 1 University of Haifa, Haifa, Israel

*Editorial Note: This report was updated on [23/09/22] to include references to works cited by the reviewer.*

 In his “Modern approached to address the concept of territorial division in Islamic jurisprudence,” H.A. Alotaibi outlines some of the contours of Muslim juristic discourses of territoriality and its association with migration. In premodern discourse, territoriality is often divided into the Abode of Islam, meaning a territory ruled by a Muslim power, and the Abode of War, meaning a territory ruled by a non-Muslim power. Alotaibi shows that the discourse of premodern jurists and modern reformers is not always so neatly divided into a binary system. What’s more, modern reformers reformulate the premodern discourse to fit an international world order governed by larger bodies such as The United Nations. After a review of the sources, the author attempts to identify a new path forward for “peaceful coexistence” and “good international relations.”

**Is the work clearly and accurately presented and does it cite the current literature?**

 The author makes it clear that some premodern and modern authors divide the Islamic world into two parts. This was well said by Khaled Abou El Fadl nearly three decades ago:

 “Premodern Muslims discussed many different types of abodes, depending on the particular objective being considered, whether territorial, theological, jurisdictional, or legal. But in all cases, it is a gross oversimplification to claim that in Islam, the world is divided into the abode of Islam the abode of infidels or war' (p190). ^1^

 The author also mentions some of the complexity of premodern discourse, but lacking from engagement is mention of just how complex the situation in premodern legal works was. A look at Abou El Fadl’s works would greatly enhance this paper. ^2^ Assumptions of a binary world divided into two territories is, as the author hints at, a rehash of orientalist views of Islam’s dichotomous worldview in which Muslims viewed Islam as in a constant state of war. But the author might have also mentioned that many modern Muslims have adopted this view without questioning its assumptions, which would help contextualize the essay and the causes of misunderstandings of the concepts of territoriality and jurisdiction.

 The article’s organization could have benefited from streamlining a thesis. By the end of the article, I was left without an idea of what contention was being made. Was there supposed to be an argument about a new model for coexistence, and if so, why was this dealt with in the short penultimate paragraph? What the article seems to be is a review of some of the issues associated with territoriality and jurisdiction. An organizational structure fitting this purpose might have been more beneficial to the reader. For example, the author might have organized around the rulings emanating from one’s of religion or the jurisdiction.  

 The paper engages the well-known and foundational texts of the major schools of Islamic law in the premodern period. There is some engagement with the texts, but many of the citations are translations without enough context or are unclear translations. For example: “Muslim law covers the people of the dhimma (non-Muslims with protected status) if the people of the heresy did not fight against the people of Sunnah, for it is the abode of Islam” (p4). The same is to be said of the translation and citation – which also includes an incomplete sentence – of Shafi’i in paragraph 2, p.9: “When contextualizing some Hadiths in this regard…” Throughout the paper, the phrase “those who…” is used abstractly without clear referents. See two examples at the beginnings of paragraphs on p.5. There are many awkward or hard transitions with unclear referents that make it difficult to follow the rationale of the statements, for example, on p.4: “The definitions of contemporaries who…”. The flow of content does not always logically follow due to a lack of signposts or organization. See for example p.6 where the author transitions to Ramadan’s assertions that appeared many paragraphs prior.

 The paper would be much improved if terms were used consistently. In the introduction, Dar al-Islam is referred to as the “land of Islam or peace” and the Dar al-Harb as the “land of war or blasphemy”. But at the end of the introduction, they are called the “house of Islam” and the “house of infidelity,” respectively; later in the translation of al-Kasani’s text the term “abode” is used. And there are more examples of inconsistency. It should also be noted that the translation of Dar al-Harb as the land of “blasphemy” does not seem to equate to the general sense of the meaning of premodern jurists. The alternate to Dar al-Harb is typically – though there are more – Dar al-Kufr or Dar al-Shirk, both meaning something like the abode of unbelief or association (with other gods), neither of which mean blasphemy. Some other better word choices might be made. Instead of “ancient” and “old” to refer to juristic activity before the modern period, “premodern” or “classical” might be used instead. For clarity, the translation of *ma’sum* and ‘ *isma *as “protection” might be termed “inviolability (of person and property).” This would be helpful to distinguish ‘ *isma* from *hirz*. Lastly, in my knowledge, the more common term used in juristic discourse for a pact of safety is Aman, as in ‘ *ahd al-aman, *rather than Amn.

 The list below notes some of the factual errors, unclear statements, and citation issues.

- P.1: “In addition, others (e.g. Alzuhaili, 1981) have added a third term, ‘Dar Al-Ahd’ (house of truce or treaty), to the existing Islamic division of the world between Dar Al-Islam and Dar Al-Harb.”
   
  - The Dar al-Ahd was spoken of since at least the 10 ^th^ century, and the way the sentence is written makes it seem as if the modern reformer created the category.
- P.8: “Secondly, contemporary jurists such as Al-Qaradawi (2010) and Topolyak (1997) have employed a new concept of “safety” to emphasize that the “house of Islam” at present is no longer confined to the historical boundaries of the old “house of Islam”. Rather, they suggest, it has expanded greatly to include every spot in which there is a Muslim who acknowledges to his Lord the two testimonies and establishes his laws in himself according to his ease and ability."
   
  - This “new” view is the same one espoused by premodern jurists who also thought that Islam is wherever a Muslim brought it, which is acknowledged in the paper (p. 7). But the paper does not cite Mawardi who made this claim. It would be helpful to show or engage the sources to clarify what is new about the view or perhaps why it mattered to the researcher or jurist being cited. It is unclear what this view adds to the development of the discourse on territoriality and jurisdiction from the premodern to the modern period.
- P9: “This is a level of magnitude unmatched in the past”.
  - This claim proceeds a statement that millions of Muslims live under non-Muslim governments. But Muslims lived at least partially under non-Muslim governance in many colonial territories, not to mention the Mongol invasion of Islamic lands in the 13 ^th^ century or the large populations of Muslims living in Sicily in the 11 ^th^, to mention just a couple examples. Some nuance would be helpful here, especially since the abode of Islam might still be considered as such even after being conquered by a non-Muslim ruler such as a Mongol. For instance, according to some in the Hanafi school, wherever Islamic law is applied, the territory is to be considered the Dar al-Islam.
- P. 9-10: “In addition, whereas Muslim people of the old abode of Islam did not leave unless they were merchants, involved in Jihad or as tourists or visitors to other places for limited periods, today, they are in many parts of the world in which Muslims permanently reside, either through long-standing affiliations to those regions or by action."
   
  - What is the basis for this contention? Is the theory of juristic discourse conflated with the fact that they were? Some proof of this contention is necessary to establish its veracity. Or alternatively it might be said that the jurists held positions that might theoretically be followed by a believer. I might suggest a review of migration studies or social history of the premodern period for explicit examples.
- P.10: “Rather, most wars in this time take place in the homes of Muslims; a scenario that did not occur in the time of the ancient jurists, as they only envisioned war between the Muslims as a whole, and the non-Muslims as a whole.”
   
  - This is difficult to understand based on other claims in the article that Orientalists such as Bernard Lewis (and Schacht might also be included here) associate a dichotomous view of the world as ones always at enmity with one another. I am not sure that the envisioning of war between Muslims and non-Muslims was of religious origin, but one of medieval societies. In the medieval period all nations were seen in conflict unless some agreement had been made. Something else to consider is whether jurists ever discuss intra-Muslim conflict, rebellion (Kharijites for example), or living under an unjust Muslim ruler.

**Are sufficient details of methods and analysis provided to allow replication by others?**

 While the article outlines some of the issues associated with premodern juristic discourse, a deeper, precise, and more substantive dive into them would have been welcome. How did the premodern law schools and their rulings on territoriality develop over time? The same could be said about the discussion of the Muslim reformers. There is mention of Abu Zahra, the twentieth-century Egyptian reformer, and al-Zuhayli, a Syrian jurist, but little to no mention is made of other well-known modern reformers (Shaltut, for example) or how they relate. Nor is there mention of the impacts of colonialism on reformers and its significance to the development of ideas of territorial boundaries in Islamic international law. Also unclear to me is the lack of specificity of the “changed nature” of international relations that resulted in reinterpretations or abrogation of premodern juristic discourse, though the author does well to mention the United Nations. Likewise, there is no discussion of premodern juristic discourse on the abrogation of the necessity for all Muslims to migrate from foreign territory to Islamic territory. All of these left me with methodological questions: why were certain modern jurists or researchers chosen as representatives of new models of juristic discourse territorial division, and how do they fit into their contexts? What were some of the contradictions in the sources that they were working out?

**Are all the source data underlying the results available to ensure full reproducibility?**

 There are references to Islamic magazines or journals that are not easily locatable and cannot therefore be accessed and analyzed by a reviewer (Alshirazi is one: the link leads to the home page). Likewise, one source citation (Eqigg) links to an unpublished PDF or produced by an unknown source that might become irretrievable. The author cites Alfaitori but does not engage his ideas. It is unclear whether Bork is a Muslim jurist, as seems evident from the article. Volume and page numbers of books should be included for reproducibility. For example, Ibn Qudama and Ibn Qayyim al-Jawziyya are cited without reference to volumes or page numbers.

**Does he fulfill his purpose?**

 The article promised to identify a new path forward for peaceful coexistence and better international relations, but they are only emphasized in a final paragraph. No specific path is identified, outlined, or examined. Instead, the article is a review of different juristic discourses, which can be found in many academic articles and books published over the last three decades or more. The article might better foregrounded a path of peaceful coexistence by relating it to the history of juristic discourse. In the end, I did not leave with a better understanding of the overlap of migration with territoriality and jurisdiction, nor of how new theories of territoriality by Muslim jurists helps the author identify a better path forward between Muslim and non-Muslim communities.

Is the work clearly and accurately presented and does it cite the current literature?

Partly

If applicable, is the statistical analysis and its interpretation appropriate?

Not applicable

Are all the source data underlying the results available to ensure full reproducibility?

Partly

Is the study design appropriate and is the work technically sound?

Partly

Are the conclusions drawn adequately supported by the results?

No

Are sufficient details of methods and analysis provided to allow replication by others?

Partly

Reviewer Expertise:

My expertise is premodern Hanbali jurisprudence. My research discusses the relations between Muslim and non-Muslims, including the rules of war and jihad, territorial boundaries and jurisdiction.

I confirm that I have read this submission and believe that I have an appropriate level of expertise to state that I do not consider it to be of an acceptable scientific standard, for reasons outlined above.

## References

[1] : Reasoning with God: Reclaiming Shari’ah in the Modern Age. *London: Rowman and Littlefield* .2014;190-190

[2] : Islamic Law and Muslim Minorities: The Juristic Discourse on Muslim Minorities from the Second/Eighth to the Eleventh/Seventeenth Centuries. *Islamic Law and Society* .1994;1(2) : 10.2307/3399332 10.2307/3399332
